# Genetic polymorphisms in homologous recombination repair genes in healthy Slovenian population and their influence on DNA damage

**DOI:** 10.2478/v10019-012-0001-7

**Published:** 2012-01-02

**Authors:** Katja Goricar, Nina Erculj, Maja Zadel, Vita Dolzan

**Affiliations:** University of Ljubljana, Faculty of Medicine, Institute of Biochemistry, Pharmacogenetics Laboratory, Ljubljana, Slovenia

**Keywords:** DNA repair, homologous recombination, genetic polymorphism, comet assay

## Abstract

**Background:**

Homologous recombination (HR) repair is an important mechanism involved in repairing double-strand breaks in DNA and for maintaining genomic stability. Polymorphisms in genes coding for enzymes involved in this pathway may influence the capacity for DNA repair. The aim of this study was to select tag single nucleotide polymorphisms (SNPs) in specific genes involved in HR repair, to determine their allele frequencies in a healthy Slovenian population and their influence on DNA damage detected with comet assay.

**Materials and methods:**

In total 373 individuals were genotyped for nine tag SNPs in three genes: *XRCC3* 722C>T, *XRCC3* -316A>G, *RAD51* -98G>C, *RAD51* -61G>T, *RAD51* 1522T>G, *NBS1* 553G>C, *NBS1* 1197A>G, *NBS1* 37117C>T and *NBS1* 3474A>C using competitive allele-specific amplification (KASPar assay). Comet assay was performed in a subgroup of 26 individuals to determine the influence of selected SNPs on DNA damage.

**Results:**

We observed that age significantly affected genotype frequencies distribution of *XRCC3* -316A>G (P = 0.039) in healthy male blood donors. *XRCC3* 722C>T (P = 0.005), *RAD51* -61G>T (P = 0.023) and *NBS1* 553G>C (P = 0.008) had a statistically significant influence on DNA damage.

**Conclusions:**

*XRCC3* 722C>T, *RAD51* -61G>T and *NBS1* 553G>C polymorphisms significantly affect the repair of damaged DNA and may be of clinical importance as they are common in Slovenian population.

## Introduction

Maintaining genetic stability is very important for survival of an individual and it requires mechanisms for repairing DNA damage that may result from exposure to heat, radiation, carcinogens and cytotoxic compounds from the environment or various endogenous metabolites.^1^

Most changes in DNA are transient because they are immediately repaired by DNA repair processes. Different types of DNA damage are recognised by different enzymes and repaired by different pathways: direct repair, excision repair and double-strand break repair. Double-strand breaks (DSBs) are a major threat to genomic stability, because if they are not repaired, they can lead to chromosome loss, chromosomal rearrangements, apoptosis or carcinogenesis.^2^ DSBs can be caused by mechanical stress, transposition and meiosis, but they also occur during DNA replication. Two pathways of DNA repair can be used for DSB repair: non-homologous end joining (NHEJ) and homologous recombination (HR). In NHEJ, long homologous templates are not required, because the ends of the break are directly ligated. Consequently, this type of repair often leads to loss of base pairs.^3^ Unlike NHEJ, HR completely repairs DSBs, results in a molecule of the same length as the original and preserves the integrity of DNA.

**FIGURE 1:**
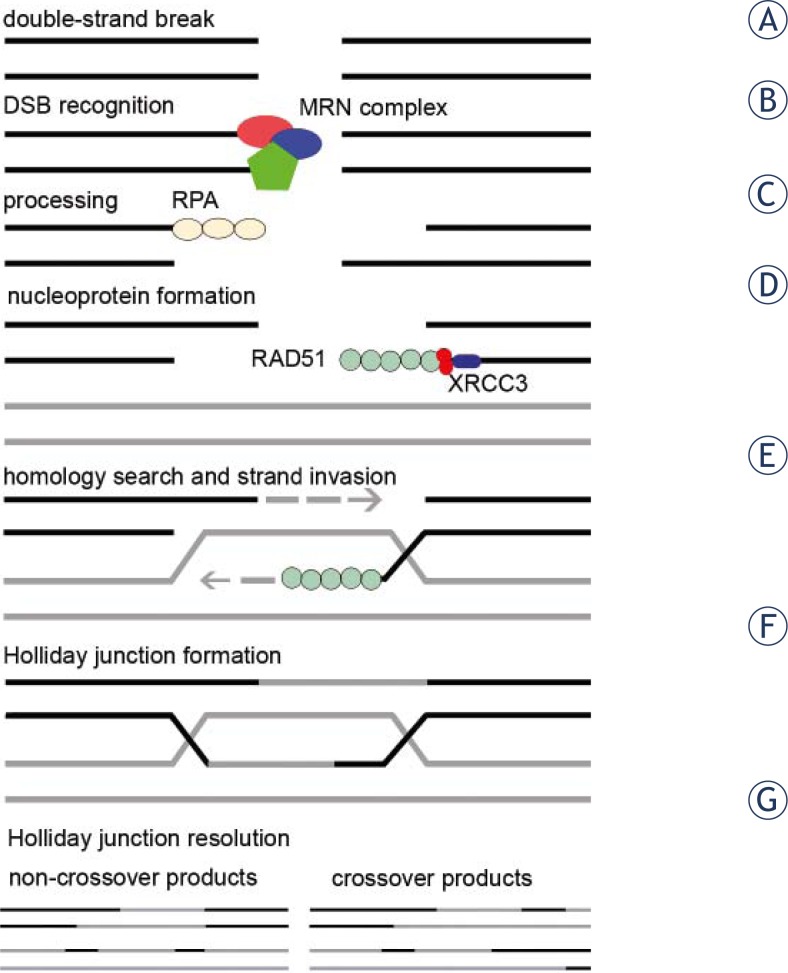
Homologous recombination repair. Double-strand breaks (A) are recognized by MRE11/RAD50/NBS1 (MRN) complex (B). The break is processed to single stranded 3′ ends, initially bound by RPA (C). With help from mediator proteins such as XRCC3, RAD51 forms a nucleoprotein filament with DNA (D). The central reaction of HR is homology search and DNA strand invasion (E), where the 3′ end of one strand invades the homologous chromatid and is elongated using the complementary strand of the homologous chromatid as a template. This results in formation of a Holliday junction with two crossovers (F). Holliday junction can be resolved in two different ways, leading to either crossover or non-crossover products, but in both cases the result of HR repair is two intact double-stranded DNA molecules (G).

HR is initiated by a DSB in one of the duplexes and the intact homologous chromosome is used as a template to repair the break by DNA synthesis as explained in Figure 1.

HR repair is a complex process, involving many different enzymes, including NBS1, RAD51 and XRCC3. NBS1 plays an important role in initial steps in both types of DSB repair as a part of MRN complex.^4^ Mutations of *NBS1* gene lead to Nijmegen breakage syndrome, presenting with immunodeficiency, increased cancer risk and radiation sensitivity.^5^ Polymorphisms in *NBS1* gene are also associated with altered cancer risk, especially in breast, lung and skin cancer.^6^ Several *NBS1* polymorphisms are relatively common, with minor allele frequency more than 5%,^6^ for example *NBS1* 553G>C (rs1805794), a non-synonymous polymorphism that changes the amino acid residue at position 185 from glutamic acid (Glu) to glutamine (Gln) (Glu185Gln), and *NBS1* 1197A>G (rs709816), a polymorphism that changes the nucleotide, but not the amino acid residue in the central region of the protein.

RAD51 catalyzes the key steps of HR repair. It possesses DNA binding and ATPase activities and interacts with several different proteins: RAD51 family, BRCA1, BRCA2 and RAD54.^7^ Several polymorphisms have been described in *RAD51* gene, although they are not common in the coding region. The polymorphisms in the 5′ untranslated region (UTR) have an important influence on gene transcription and protein expression. Among them, *RAD51* -98G>C (rs1801320) and *RAD51* -61G>T (rs1801321) were reported to increase promoter activity.^8^ Polymorphisms in the 3′ UTR, such as *RAD51* 1522T>G (rs12593359), may play an important role in regulation of gene expression by controlling polyadenylation, translation rate and mRNA degradation.^9^

XRCC3 is a member of RAD51 family and one of *XRCC* genes that help protect the cell from the effects of ionizing radiation.^10^ The *XRCC3* gene region contains mostly intronic polymorphisms, however a few polymorphisms that change amino acid residues were described in the coding region, but their impact is largely unknown.^11^ Most studies have investigated the *XRCC3* 722C>T (rs861539) polymorphism that leads to the substitution of threonine (Thr) with methionine (Met) at position 241 (Thr241Met) and *XRCC3* -316A>G (rs1799794) polymorphism in 5’ UTR.

Single nucleotide polymorphisms (SNPs) are the most common form of DNA variation. SNPs in genes coding for enzymes involved in DNA repair can modify the activity or expression of these enzymes and change the DNA repair capacity, which can, in turn, result in increased risk for various diseases, especially cancer. They can also influence cancer treatment response and efficacy.^12^ As they may vary considerably among different populations, it is important to identify the frequencies of these SNPs in the population. Furthermore, SNPs can be used as pharmacogenetic markers when their functional significance and the association with a given phenotype are proven.^13^ Instead of functional SNPs, the tagged SNP approach can be used to cover the variability within the gene region. Based on haplotype information available from the International HapMap Project database (http://www.hapmap.org) we can select only one tag SNP from an area with high linkage disequilibrium without loss of information.

The aim of this study was to select tag SNPs in *XRCC3*, *RAD51* and *NBS1* genes and determine their frequencies in a healthy Slovenian population. We also wished to assess their influence on DNA damage detected with comet assay and evaluate the impact of age on genotype frequencies.

## Materials and methods

### Participants

The study population consisted of 373 healthy Slovenian blood donors. A randomly selected subgroup of 26 individuals was invited to participate in comet assay. All subjects in the subgroup were asked to refrain from physical activity for at least two days before venepuncture. We also obtained data on smoking and folate intake from this subgroup, using a questionnaire. Daily folate intake was calculated using the program Alimentación y Salud 2.0. Written informed consent was obtained from all individuals prior to participation. The study was approved by the Slovenian Ethics Committee for Research in Medicine and was carried out according to the Helsinki Declaration.

### Bioinformatic analysis

We used public databases to determine the tag SNPs in genes for three enzymes that are part of homologous recombination repair: XRCC3, RAD51 and NBS1. We searched for the data on polymorphisms in these genes in the International HapMap Project database (http://www.hapmap.org), checked the location and allele frequencies of SNPs in the Caucasian population in the SNP database (www.ncbi.nlm.bih.gov/projects/SNP) and selected the tag SNPs using HaploView (http://www.broad.mit.edu/haploview/haploview-downloads#DOWNLOAD).

Next, we determined which SNPs are genetically linked and therefore form haplotype blocks. We set the r^2^ value to 0.8, meaning there was an 80% chance for SNPs in the haplotype block to be genetically linked. From each haplotype block we chose at least one tag SNP, preferentially a SNP that had been previously investigated and had an impact on protein expression or stability, or changed amino acids. We selected only SNPs with minor allele frequency higher than 0.050 (more than 5% of the population).

### Genotyping

Genomic DNA was isolated from peripheral venous blood leukocytes using a Qiagen FlexiGene kit according to the manufacturer’s recommendations (Qiagen, Hilden, Germany). DNA concentration was determined by absorbance measurements.

Competitive allele-specific amplification (KASPar assay, KBioscience, Hoddesdon, Herts, UK) was used for genotyping selected polymorphisms. This assay uses a pair of allele-specific primers and a pair of probes. Allele-specific primer binds to target DNA sequence at the 3′ end, but has a 5′ tail sequence that is not complementary to the target sequence. A different probe binds to the tail sequence of each allele-specific primer. Each probe consists of a region, complementary to the respective tail sequence, and a stem loop structure with a different fluorophore and a quencher. During PCR, the allele - specific primer is elongated and the tail sequence becomes incorporated in the PCR product. One of the probes hybridizes with the respective tail sequence and is also coupled to the PCR product. During the next PCR cycle, the stem loop is linearized and the termination of quenching effect results in a fluorescence signal. The wavelength of the signal is probe specific and depends on which allelic variant is present in the sample.^14^

We performed the amplifications in GeneAmp PCR System 9700 AB (Applied Biosystems, Foster City, California, USA) as recommended by the manufacturer (KBioscience). We measured the fluorescence on a 7500 Real Time PCR System AB and analysed the data with 7500 System SDS Software (both Applied Biosystems).

### Comet assay

Comet assay (also called single-cell gel electrophoresis), a simple and sensitive method for detecting single and double-strand breaks,^15^ was used to assess DNA damage in lymphocytes from 26 healthy individuals as previously described.^16^ Comet assay was performed blind to the genotyping data. In brief, lymphocytes embedded in low melting point agarose were lysed and subjected to unwinding and electrophoresis under alkaline conditions.^17^ After staining with a DNA-binding dye, DNA damage was visualised by fluorescence microscopy and quantified using Comet 5 software (Kinetic Imaging Ltd, 2000, UK). The level of DNA damage was evaluated by two parameters: the percentage of DNA in the tail (% TD) and the Olive Tail Moment (OTM). OTM is the product of the % TD and tail length.

### Statistical analysis

The median was used to present central tendency of normally distributed parameters, while the range (minimum-maximum) was a measure of variability. To describe non-normally distributed parameters mean values and standard deviation (SD) were used. A chi-square statistic was used to verify that allele frequencies were in Hardy-Weinberg equilibrium (HWE). In all statistical analysis the dominant genetic model was used, which specifically tests the associations of having at least one minor allele versus having two wild type alleles. We tested the normality of variables’ distribution with the Shapiro-Wilk test. We used nonparametric correlations to compare genotype frequency distributions between the age groups. As % TD and OTM were not normally distributed, we used nonparametric Mann-Whitney U-test to determine the influence of polymorphisms on DNA damage. Multivariable linear regression was used to determine the influence of studied genetic polymorphisms on OTM using normally distributed logarithmic values of OTM. All non-genetic variables that had an influence on DNA damage were included in the multivariable model. The level of significance for all tests was set to P < 0.050. All statistical analyses were performed using SPSS for Windows 14.0.1 software (Statistical Package for the Social Sciences, Chicago, IL).

## Results and discussion

The study group consisted of 373 healthy Slovenian subjects, 219 (58.9%) male and 153 (41.1%) female with a median age of 30 (range 18–65) years.

In our study we first selected the tag SNPs in three genes that have an important role in HR repair. Most of the genetic variability within the respective gene regions was covered with two tag SNPs for *XRCC3*, three SNPs for *RAD51* and four SNPs for *NBS1*. The distribution of genotype frequencies for selected tag SNPs is summarized in Table 1. All genotype distributions were consistent with HWE (P > 0.050).

The observed genotype frequencies in the Slovenian population were mostly in agreement with values published for other Caucasian populations in the SNP database (dbSNP) and other studies.^8^,^18^–^21^ The distribution of genotype frequencies among populations differed significantly only for *NBS1* 553G>C polymorphism, as shown in Table 2. Polymorphic *NBS1* 553C allele was the common allele both in our study and in dbSNP, while the normal *NBS1* 553G allele was reported to be more common in Eastern European populations.^20^–^22^

To determine whether age affected genotype distribution for selected tag SNPs, we divided the subjects into ten five-year age groups (18–22 years, 23–27 years, 28–32 years, 33–37 years, 38–42 years, 43–47 years, 48–52 years, 53–57 years, 58–62 years and 63–65 years). Because the age groups had a considerably different sex distributions (P < 0.001) and *XRCC3* 722C>T genotype frequencies were differently distributed between males and females (P = 0.007), we determined the impact of age on genotype frequency distribution only in men.

**FIGURE 2:**
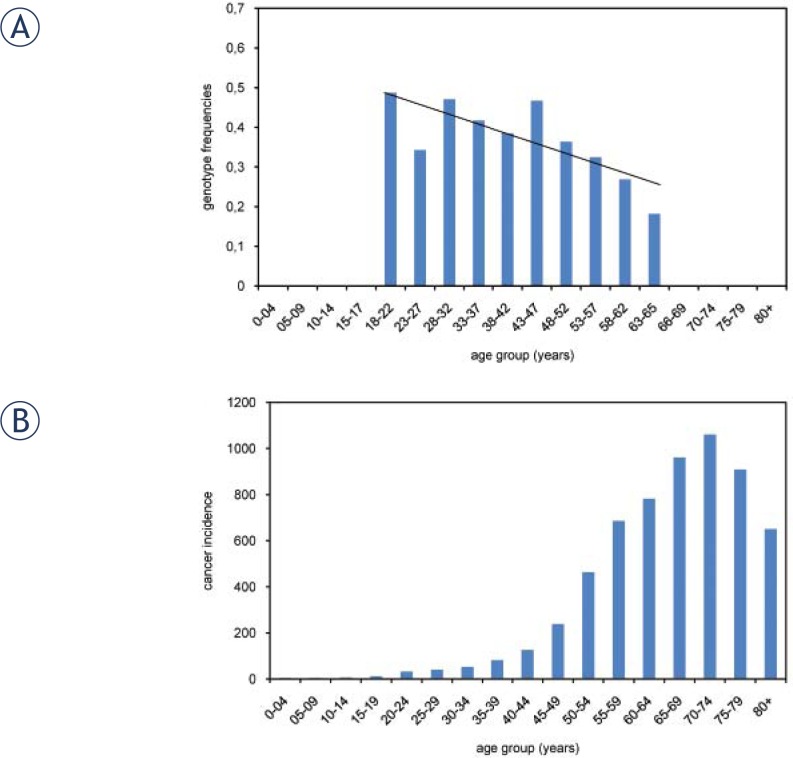
*XRCC3* -316A>G polymorphism and age. Proportion of men with polymorphic *XRCC3* -316G allele decreases with increasing age, especially above the age of 50 years (A). Cancer incidence in Slovenia for men in 2007. Cancer incidence significantly increased above the age of 50 years (B).

The nonparametric correlations were used to determine if the genotype frequencies differ among the age groups. A statistically significant difference was observed only for the frequency distribution of *XRCC3* -316A>G polymorphism (Kendall’s tau = -0.121, P = 0.039). With increasing age, the percentage of individuals carrying the polymorphic allele decreased, most notably above the age of 50 years (Figure 2A). As this age coincides with a rapid increase in cancer incidence in the Slovenian population (Figure 2B),^23^ the loss of the polymorphic *XRCC3* -316G allele from the healthy population with increasing age may indicate that this or another closely linked SNP influences DNA repair capacity and plays a role in cancer risk. Previous studies did not find a direct association between this polymorphism and cancer risk^19^, however a recent study showed an association of polymorphic *XRCC3* -316GG genotype with worse prognosis and survival in gastric and oesophageal cancer.^24^

We also investigated the influence of selected SNPs in genes coding for HR repair enzymes on level of DNA damage. DNA damage was determined by the comet assay in a subgroup of 26 individuals. This subgroup consisted of 20 female and 6 male individuals with median age of 24 (range 20 – 29) years. The values for folate intake varied between 41.5 and 645.1 μg folate per day (mean value 327.46 ± 167.90 μg per day). Three individuals in the subgroup were smokers. DNA damage was quantified as % TD and OTM. The mean % TD (±SD) was 7.40 (± 2.36) and mean OTM (±SD) was 0.99 (± 0.56). Even though the % TD is linearly related to DNA damage, we chose OTM as a better marker of DNA damage, because we only investigated healthy individuals and we expected a low degree of DNA damage. In such a case the most informative parameter is tail length, which is used to calculate OTM.^25^

Because some studies reported that smoking influenced the level of DNA damage,^26^,^27^ while others observed no influence,^28^ we checked for the influence of smoking in our subgroup. Similar to the results of the latter study, we did not observe any association between smoking and OTM (P = 0.507), so we did not exclude the smokers from further analysis.

We analysed the influence of polymorphisms on DNA damage using nonparametric tests and the results are summarized in Table 3. The variability of the average OTM between genotypes was rather low in general. Although none of the investigated polymorphisms had a statistically significant effect on % TD (P≥0.050 for all associations), *XRCC3* 722C>T (P = 0.004), *RAD51* -61G>T (P = 0.034) and *NBS1* 553G>C (P = 0.002) had a statistically significant influence on OTM in univariable analysis.

We also determined the influence of non-genetic factors on DNA damage in univariable regression analysis. Sex and age did not significantly influence OTM (P = 0.969 and P = 0.069, respectively), but folate intake had a statistically significant influence on OTM (P = 0.048). Only polymorphisms *XRCC3* 722C>T, *RAD51* -61G>T and *NBS1* 553G>C that had a statistically significant influence on DNA damage were included in multivariable regression model adjusted for age and folate intake. Because the variables must be normally distributed for this analysis, we used the normally distributed logarithmic values of OTM (P > 0.050 in Shapiro-Wilk analysis). Even though all P values were not statistically significant (P > 0.050), all variables included in the regression model contributed to R^2^ value (R^2^ = 0.565). As shown in Table 4, only the influence of the *XRCC3* 722C>T polymorphism on DNA damage remained significant in this model.

Because *XRCC3* 722C>T, *NBS1* 553G>C and *RAD51* -61G>T have a functional effect, it is not surprising that they influence DNA damage. Several studies have investigated the impact of these polymorphisms on cancer risk, but the results are inconsistent.

The *NBS1* 553G>C polymorphism changes the amino acid in the BRCA1 C-terminal domain, important for interaction with histones and relocalisation of the MRN complex closer to DNA damage.^29^ It could therefore affect interaction with other proteins that are part of HR repair. In comet assay, individuals with *NBS1* 553GG genotype had a higher OTM value than individuals with 553GC or 553CC genotypes. A previous study reported that this polymorphism modulates the frequencies of chromatid-type aberrations^30^ and some other studies investigated the impact of this polymorphism on cancer risk. One study reported that polymorphic allele increased the risk for basal cell carcinoma in males^21^, but no association was found between this polymorphism and breast cancer^19^ or acute lymphoblastic leukaemia risk.^20^

*XRCC3* 722C>T polymorphism leads to amino acid substitution, which could affect the protein structure or function. In the present study, individuals with *XRCC3* 722CC genotype had higher OTM value than carriers of 722T allele. Previous studies that investigated the influence of *XRCC3* 722C>T polymorphism on chromosomal aberrations and single-stranded breaks did not report any influence of this polymorphism on DNA damage.^31^ Several studies investigated the effect of this polymorphism on cancer risk, but the results were also inconclusive.^18^,^19^,^21^,^32^,^33^ Most studies link this polymorphism with breast cancer risk, where homozygotes for the polymorphic allele have an increased risk compared to homozygotes for the normal allele.^19^ The polymorphic allele was also associated with increased risk of lung cancer.^32^ In contrast with these studies, no influence on the risk of stomach cancer was found^18^ and the polymorphic allele was even associated with a reduced risk of basal cell carcinoma.^21^ A meta-analysis that compared the findings of different studies concluded that polymorphisms in *XRCC3* gene definitely modify the risk, especially for some types of cancer, but do not represent the most important risk factor.^33^

*RAD51* -61G>T modifies promoter activity and the polymorphic allele facilitates binding of a transcription factor, thus increasing the transcription of the gene.^8^ In our study, individuals with *RAD51* -61GG genotype had a higher OTM value than individuals with -61GT or -61TT genotypes. It was also established that cells with *RAD51* -61TT genotype had fewer gamma radiation-induced chromatid breaks^8^, which is consistent with the proposed protective effect of this genotype. These results suggest that the polymorphic allele is associated with a smaller amount of DNA damage. Some studies also investigated the influence on cancer risk and a decreased risk was observed for squamous cell carcinoma of the head and neck among homozygotes for the polymorphic allele^8^ and for breast cancer among the carriers of the polymorphic *RAD51* -61T allele.^19^

We report the results of the first study investigating polymorphisms in genes for HR repair enzymes in a Slovenian population and their influence on DNA damage. Our study included subjects from an ethnically homogenous population, which is important because the association between genotype frequencies and risk of cancer can differ among populations.^34^ Because the participants in our study were blood donors, we could not include people older than 65 years. This could be a limitation when assessing the influence of age on genotype frequencies, but we were able to show that age significantly affects genotype frequencies already above the age of 50 years, which coincides with a significant increase of cancer incidence. The main limitation of our study though was the small number of individuals included in the subgroup for analysis of DNA damage with comet assay. Despite this limitation, we managed to show that three selected tag SNPs had a statistically significant influence on DNA damage. Because they are very common in Slovenian population, they are of major interest for further pharmacogenetic studies, investigating the association between DNA repair polymorphisms and cancer risk or cancer treatment response as a part of generic testing which became extremely important in oncology.^35^

## Figures and Tables

**TABLE 1:** Genotype frequencies of selected tag SNPs in healthy individuals (N = 373)

**Polymorphism**	**SNP rs number**	**Genotype frequencies N (%)**		
*XRCC3* 722C>T (Thr241Met)	rs861539	CC	CT	TT
		153 (41.0)	158 (42.4)	62 (16.6)
*XRCC3* -316A>G	rs1799794	AA	AG	GG
		247 (66.2)	107 (28.7)	19 (5.1)
*RAD51* -98G>C	rs1801320	GG	GC	CC
		304 (81.5)	61 (16.4)	8 (2.1)
*RAD51* -61G>T	rs1801321	GG	GT	TT
		133 (35.7)	176 (47.2)	64 (17.2)
*RAD51* 1522T>G	rs12593359	TT	TG	GG
		103 (27.6)	176 (47.2)	94 (25.2)
*NBS1* 553G>C (Glu185Gln)	rs1805794	GG	GC	CC
		33 (8.8)	180 (48.3)	160 (42.9)
*NBS1* 1197A>G Asp399Asp)	rs709816	AA	AG	GG
		139 (37.3)	188 (50.4)	46 (12.3)
*NBS1* 37117C>T	rs1805811	CC	CT	TT
		347 (93.0)	25 (6.7)	1 (0.3)
*NBS1* 3474A>C	rs1063054	AA	AC	CC
		163(43.7)	170 (45.6)	40 (10.7)

SNP, single nucleotide polymorphism

**TABLE 2:** The distribution of genotype frequencies for NBS1 553G>C polymorphism in different Caucasian populations

**Genotype frequencies (%)**						

**Population**	**N**	**GG**	**GC**	**CC**	**P**	**Reference**
Slovenia	373	8.8	48.3	42.9	-	present study
HapMap-CEU	120	8.3	40.0	51.7	0.233	dbSNP
United Kingdom	734	10.5	43.3	46.2	0.272	Kuschel *et al.*^19^
Poland	275	40.4	48.7	10.9	< 0.001	Mosor *et al.^20^*
Hungary, Romania, Slovakia	533	46.9	41.5	11.6	< 0.001	Thirumaran *et al.^21^*
Czech Republic	530	45.1	41.5	13.4	< 0.001	Pardini *et al.^22^*

HapMap-CEU, population with European ancestry, included in HapMap project

**TABLE 3:** The influence of selected polymorphisms on DNA damage detected with comet assay in a subgroup of 26 individuals

**Polymorphism**	**Genotype**	**N (%)**	**OTM (mean ± SD)**	**P**
*XRCC3* 722C>T	CC	13 (50.0)	1.192 ± 0.706	0.004
	CT+TT	13 (50.0)	0.795 ± 0.280	
*XRCC3* -316A>G	AA	22 (84.6)	1.002 ± 0.607	0.663
	AG+GG	4 (15.4)	0.948 ± 0.253	
*RAD51* -98G>C	GG	22 (84.6)	1.019 ± 0.606	0.856
	GC+CC	4 (15.4)	0.853 ± 0.220	
*RAD51* -61G>T	GG	7 (26.9)	1.419 ± 0.946	0.034
	GT+TT	19 (73.1)	0.837 ± 0.221	
*RAD51* 1522T>G	TT	8 (30.8)	0.779 ± 0.179	0.173
	TG+GG	18 (69.2)	1.089 ± 0.650	
*NBS1* 553G>C	GG	4 (15.4)	1.355 ± 0.224	0.002
	GC+CC	22 (84.6)	0.9277 ± 0.585	
*NBS1* 1197A>G	AA	6 (23.1)	0.778 ± 0.127	0.290
	AG+GG	20 (76.9)	1.058 ± 0.629	
*NBS1* 37117C>T	CC	23 (88.5)	1.018 ± 0.594	0.600
	CT+TT	3 (11.5)	0.8033 ± 0.170	
*NBS1* 3474A>C	AA	10 (38.5)	0.783 ± 0.148	0.132
	AC+CC	16 (61.5)	1.125 ± 0.685	

SD, standard deviation

**TABLE 4:** The influence of genetic and non-genetic factors on DNA damage

**Variable**	P*
Constant	0.126
Age	0.258
Folate intake	0.103
*XRCC3* 722C>T	0.040
*RAD51* -61G>T	0.122
*NBS1* 553G>C	0.129

* in multiple regression model for logarithmically transformed OTM values
